# EAT-Lancet reference diet and nutritional adequacy in children: examining the planetary health diet index for children (PHDI-C)

**DOI:** 10.1007/s00394-025-03858-9

**Published:** 2025-12-19

**Authors:** Elise Fabios, Itziar Zazpe, Susana Santiago, Silvia García, Miguel Ángel Martínez-González, Nerea Martín-Calvo

**Affiliations:** 1 Department of Preventive Medicine and Public Health, School of Medicine, University of Navarra, Pamplona, Spain; 2 Department of Nutrition, Food Science and Physiology, School of Pharmacy, University of Navarra, Pamplona, Spain; 3 Biomedical Research Centre Network on Obesity and Nutrition (CIBERobn), Physiopathology of Obesity and Nutrition, Institute of Health Carlos III, Madrid, Spain; 4 IdiSNA, Instituto de Investigación Sanitaria de Navarra, Pamplona, Spain; 5 Research Group on Community Nutrition and Oxidative Stress, University of the Balearic Islands-IUNICS, Palma de Mallorca, Spain; 6 Health Research Institute of the Balearic Islands (IdISBa), Palma de Mallorca, Spain

**Keywords:** EAT-Lancet diet, Planetary health diet index, Micronutrient adequacy, PHDI-C, SENDO Cohort

## Abstract

**Purpose:**

The EAT-Lancet Commission introduced a Planetary Health Diet (PHD) in 2019 for individuals aged two years and older. Concerns exist regarding its ability to fulfill children’s specific micronutrient requirements. The PHDI-C, an adapted version of the Planetary Health Diet Index (PHDI), was developed to better reflect the nutritional needs of growing children. However, its association with micronutrient adequacy in pediatric populations has not been fully examined. Our study investigates whether the PHDI-C is associated with micronutrient adequacy in children and compares its performance to the original PHDI.

**Methods:**

This cross-sectional study compared the association of the PHDI and PHDI-C with nutritional adequacy in 945 children aged 4–5 years from the SENDO cohort. Dietary, lifestyle, and socio-demographic data were collected via parent-administered questionnaires. Dietary intake was assessed using a Food Frequency Questionnaire (FFQ). Scores for both indices were calculated and ranged from 0 to 150, and participants were categorized into tertiles. We calculated the intake of 20 micronutrients relevant to public health and micronutrient adequacy was assessed using the Estimated Average Requirement (EAR) cut-off points. Generalized estimating equation models were used to examine the relationship between both indices and the risk of inadequate micronutrient intake.

**Results:**

Median scores were 63.0 and 62.3 for the PHDI and PHDI-C respectively. Higher PHDI and PHDI-C scores were associated with higher intakes of vitamins A, C, E, B1, B3, and B6, as well as folate, Fe, Mg, Se, Zn, Cr, and K, but lower intakes of vitamins B2, B12, and Ca, and I. After adjusting for potential confounders, higher PHDI-C scores were associated with fewer unmet EARs *(p* < *0.001).* Children in the highest tertile of the PHDI-C had 55% lower odds (OR:0.45 95% CI: 0.22–0.94) of having ≥ 3 inadequate micronutrient intakes compared to the lowest tertile. For the original PHDI, children in the highest tertile had 30% lower odds (OR: 0.70; 95% CI: 0.32–1.54).

**Conclusion:**

The PHDI-C is linked to better nutritional adequacy than the original PHDI. Further research is needed to validate the PHDI-C in terms of environmental sustainability.

**Supplementary Information:**

The online version contains supplementary material available at 10.1007/s00394-025-03858-9.

## Introduction

The human diet has undergone significant transformations in recent decades due to technological advancements, globalization, and shifts in agricultural practices. However, the current food system poses a threat to both human and planetary health, contributing to unhealthy dietary habits and the depletion of natural and environmental resources [1, 2]. To tackle these challenges, the EAT-Lancet Commission introduced the “Planetary Health Diet” (PHD) in 2019—a reference diet designed to improve human health while promoting environmental sustainability. Its innovation and strength lie in the development of scientific targets, grounded on the best available evidence, relating to dietary patterns, health outcomes, and ecological sustainability [3]. The reference diet is intended for individuals aged two years and older and emphasizes a large proportion of vegetables, fruits, whole grains, legumes, nuts, and unsaturated oils. It includes a low to moderate amount of seafood, eggs, and poultry while minimizing red meat, processed meat, added sugars, refined grains, and starchy vegetables [3].

Researchers have developed several indices to measure adherence to the EAT-Lancet diet [4, 5] and its effects on health [5–11]. However, when it comes to nutrient adequacy, a key aspect of diet quality, evidence remains scarce [12–14]. Concerns have been raised about the diet’s low intakes of animal-source foods and the bioavailability of certain plant-based nutrients, which may make it difficult to meet micronutrient needs, especially in those subgroups of the population who have different dietary requirements [15–18]. Notably, in young children, no study has directly assessed the relationship between the EAT-Lancet diet and micronutrient adequacy.

Building on this context, Venegas et al. adapted the published index (i.e., the Planetary Health Diet Index (PHDI) proposed by Cacau et al.[19], and developed the new Planetary Health Diet Index for Children and Adolescents (PHDI-C) [20], which is better tailored to the nutritional needs of children and adolescents. Both the PHDI and PHDI-C use energy-adjusted cut-off values, allowing to account for age-specific energy and nutrient requirements. Additionally, these indices apply continuous scoring scales rather than dichotomous categorizations, thus enhancing their discriminant abilities [21]. Key modifications from the PHDI to the PHDI-C include a) modification of food group thresholds to better accommodate children’s nutritional needs (mainly dairy products, eggs and white meats); b) inclusion of refined cereals and a whole cereals-to-total cereals ratio to balance micronutrient bioavailability and account for phytate levels; and c) the separation of palm oil from other vegetable oils to better align with EAT-Lancet dietary recommendations.

The objectives of our study were to evaluate the validity of this new index for measuring nutritional adequacy and to compare its performance with the original PHDI in young children.

## Material and methods

### Study population

The SENDO Project is a dynamic, prospective cohort study of Spanish children that started participant recruitment in 2015 (https://www.proyectosendo.es/). Its main objective is to evaluate the impact of lifestyle and dietary habits on the health of children and adolescents. Participants are invited to join the cohort through their pediatrician at healthcare centers or via the SENDO research team collaborating with schools. Eligibility criteria include being 4 or 5 years old and residing in Spain. The only exclusion criterion is the inability to access an internet-connected device required to complete online questionnaires. Parents complete these self-reported questionnaires at baseline, and then yearly. The baseline questionnaire collects data on medical history, anthropometric measures, diet, lifestyle, and sociodemographic variables.

This cross-sectional study used baseline data from 1328 children recruited between January 2015 and June 2024. We excluded 165 with extreme energy (< P1 or > P99) and an additional 125 participants with extreme micronutrient intakes (≥ + 3 or ≤ −3 standard deviations (SD)), respectively. Finally, 93 additional children were excluded due to incomplete baseline questionnaires. The final sample comprised 945 participants.

### Ethical approval

The SENDO project complies with the ethical principles outlined in the Declaration of Helsinki for medical research involving human subjects. The study received approval from the Ethics Committee for Clinical Research of Navarra (P. 2016/122). Informed consent was obtained from all participants’ parents during recruitment.

### Dietary assessment

Information on usual dietary intake was gathered using a validated semi-quantitative food-frequency questionnaire (FFQ) consisting of 147 items [22], completed by the parents. A standard portion size was specified for each food item. Parents reported how often their child had consumed each item over the previous year by selecting one out of nine frequency categories, ranging from “never or almost never” to “ ≥ 6 times per day”. A team of trained dietitians determined the nutrient content of each food by multiplying intake frequency by the edible portion and the nutrient composition of the specified portion size. To estimate total energy and nutrient intake, we used updated Spanish food composition tables [23] and online databases [24, 25], summing the contributions of all food items recorded in the FFQ.

We also evaluated dietary intake based on 15 distinct food categories, including vegetables, fruits, legumes, grains, potatoes, nuts, dairy products, eggs, fish and seafood, meat, fast-food, pastries and sweets, sugar-sweetened beverages, olive oil, and other vegetable oils.

### EAT-Lancet scores: PHDI and PHDI-C

We calculated adherence to the EAT-Lancet diet using the PHDI, based on the article by Cacau et al. and the PHDI-C, as proposed by Venegas et al. The scoring criteria for both indices are shown in Table 1 and have been described in previous studies [19, 20].

**Table 1:**
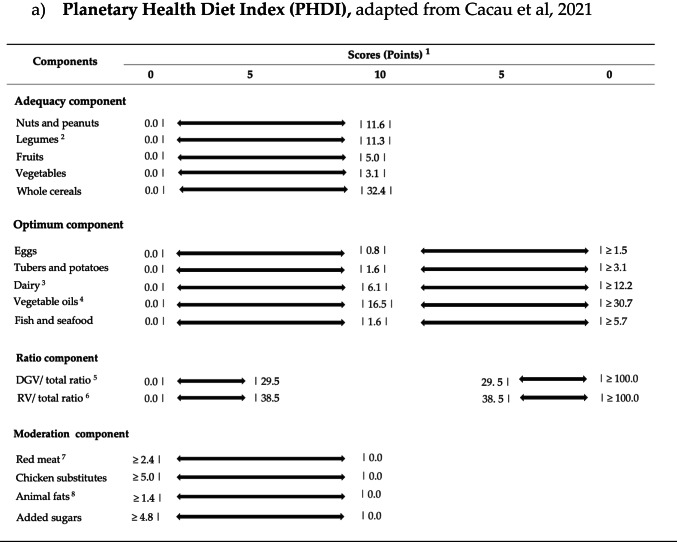

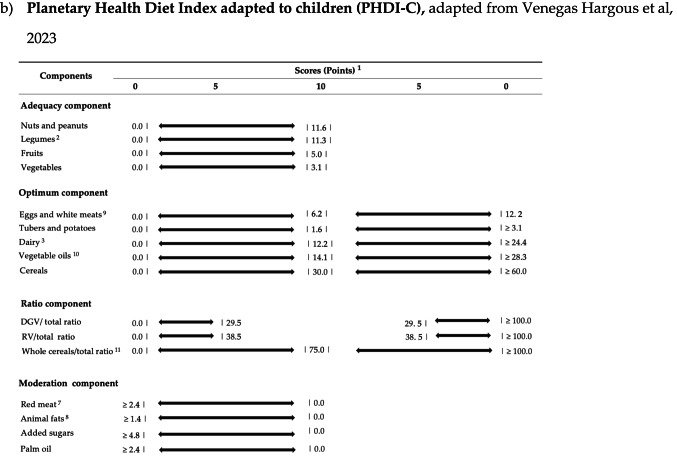
Components, standards for scoring (caloric densities) and corresponding point values of the planetary health diet index and the planetary health diet index adapted for children

1 All values expressed as caloric densities from the reference diet proposed by the EAT-Lancet Commission. The bars represent the limits; 2 Legumes: beans, lentils and soy; 3 Dairy: excluding dairy fats; 4 Unsaturated oils (includes palm oil); 5 DGV ratio: ratio between the energy intake of dark green vegetables and the total of vegetables; 6 RV ratio: ratio between the energy intake of red and orange vegetables and the total of vegetables; 7 Red meat: beef, lamb, and pork; 8 Animal fat: lard, tallow and dairy fats; 9 Eggs and white meats: includes eggs, fish, seafood and chicken; 10 Unsaturated oils (does not include palm oil)

For the PHDI, the score is categorized into 16 items, grouped into 4 main components: adequacy, optimum, ratio, and moderation. The adequacy component includes foods recommended in high quantities, with no negative scoring associated with higher consumption. The optimum component consists of foods with specific intake ranges, where intakes falling below or above the thresholds results in diminishing positive scoring. The ratio component accounts for the variety of vegetable intake and also results in diminishing positive scoring when intakes fall below or above the specified threshold. The moderation component includes foods that should be limited or avoided entirely, where no consumption receives maximum score.

Key modifications made to the PHDI in developing the PHDI-C include: a higher threshold for dairy intake, allowing for more dairy consumption without negative scoring to ensure sufficient Calcium (Ca) and vitamin D intake; grouping eggs, poultry, and fish together to better reflect children’s protein needs; adjusting whole cereal intake to balance nutrient bioavailability and fiber intake. Whole grains were replaced by “Grains” in the adequacy component, but a whole cereals-to-total cereals ratio (75%) was introduced to account for whole grain consumption**;** separating palm oil from other unsaturated vegetable oils to better reflect its health impacts. The total score for both indices ranges from 0 to 150 points.

### Extracting PHDI components from food consumption data

Energy intake for each index component was calculated following the procedure described in the PHDI validation study [19]. Each of the 147 food items in the FFQ was assigned to the appropriate index component (food group), and daily intakes (g/day) were summed, converted to energy (kcal), and expressed as the percentage of total energy intake. This caloric density was used to score each component according to the predefined thresholds of the index.

Mixed dishes and processed products were decomposed into individual ingredients using national household standard recipes [26], for later classification into the food groups used to calculate the dietary scores. For highly processed foods dominated by a single ingredient (e.g., maize-based chips), total energy was allocated to ingredient groups according to added sugar and fat content from the nutrient database—for example, fat calories were attributed to vegetable oils and the remainder to refined cereals. For products widely available on the market, ingredient composition was averaged across two or three leading brands or typical product types. Processed meats were not decomposed but classified by primary ingredient into the red meat group (e.g., sausage, ham, salami) or the chicken and substitutes group (e.g., pâté, nuggets).

### Covariates

Parents provided sociodemographic information about the participants. Physical activity data was collected through a questionnaire covering 14 types of activities, with 10 response options ranging from “never” to “ ≥ 11 h per week.” Participants reported the average time spent on each activity over the previous year. We calculated the annual mean hours per day spent in moderate-vigorous activities. Screen time was measured by calculating the average daily hours spent using screens (TV, computers, or video games), with separate assessments for weekdays and weekends.

Parental attitudes toward their child’s dietary habits were evaluated using 8 yes/no questions, where healthy attitudes received a positive score, and unhealthy ones received no points. Parental knowledge of nutritional recommendations for children was assessed through questions on the recommended intake frequency of 10 food groups. Detailed descriptions of these indices can be found in prior publications [27, 28].

### Outcome assessment

The intake of 20 micronutrients was evaluated, including vitamins A (measured as retinol equivalents), C, D, E, B1, B2, B3, B6, B12, folate, Ca, iodine (I), iron (Fe), phosphorus (P), magnesium (Mg), selenium (Se), zinc (Zn), chromium (Cr), potassium (K), and sodium (Na). Micronutrient intake was classified as inadequate if it fell below the estimated average requirement (EAR), using the Harmonized Nutrient Reference values defined by Allen and colleagues [29]. For nutrients lacking an EAR (Na and K), Adequate Intake (AI) values were applied. As widely recognized in nutritional science, a median intake above the AI indicates adequacy, whereas a median below the AI does not allow a definitive conclusion.

### Statistical analysis

Participants were grouped into tertiles based on their PHDI and PHDI-C scores, with the highest tertile representing the highest adherence. For descriptive purposes, categorical variables were summarized as numbers and percentages, while continuous variables were presented as means and standard deviations (SD). Linear trend tests across tertiles were conducted by assigning the median value of each tertile and treating this as a continuous variable in regression models.

We also assessed the energy-adjusted intake of micronutrients in each tertile and the energy-adjusted increase in micronutrient intake for every 10-point increase of the PHDI and PHDI-C scores. Energy-adjusted intakes of micronutrients were obtained through regression models.

To evaluate the relationship between both indices and the risk of  ≥ 3 inadequate intakes, we used the EAR cut-point method and fitted generalized estimating equations to account for intra-cluster correlation among siblings (91 siblings in our final sample). Multivariate models were progressively adjusted for: 1) sex, age (years), and total energy intake (kcal); 2) breastfeeding duration (none, < 6, 6–12, and > 12 months), parental knowledge of nutritional recommendations for children (low, medium and high) and parental attitudes towards child’s dietary habits (unhealthy, average and healthy); 3) physical activity (h/day of moderate-vigorous activities) and screen time (h/day). The first tertile was always used as the category of reference. The analysis consisted of calculating: 1) the difference and 95% Confidence Interval (CI) in the number of inadequate intakes of micronutrients across values of the PHDI and PHDI-C and 2) the Odds ratios (ORs) and 95% CIs for the inadequate intake of ≥ 3 micronutrients. Multivariable-adjusted proportions of children with ≥ 3 inadequate micronutrient intakes were also estimated for each tertile of both indices.

We also performed a sensitivity analysis, similar to that of Lassen and colleagues [30], using 1400 kcal as a reference intake for this age range and calculating micronutrient intakes proportionally.

All statistical analyses were performed using Stata version 15.0 (Stata Corp., College Station, TX, USA). Two-tailed p-values were used, with statistical significance set at *p* < 0.05.

## Results

**Fig. 1:**
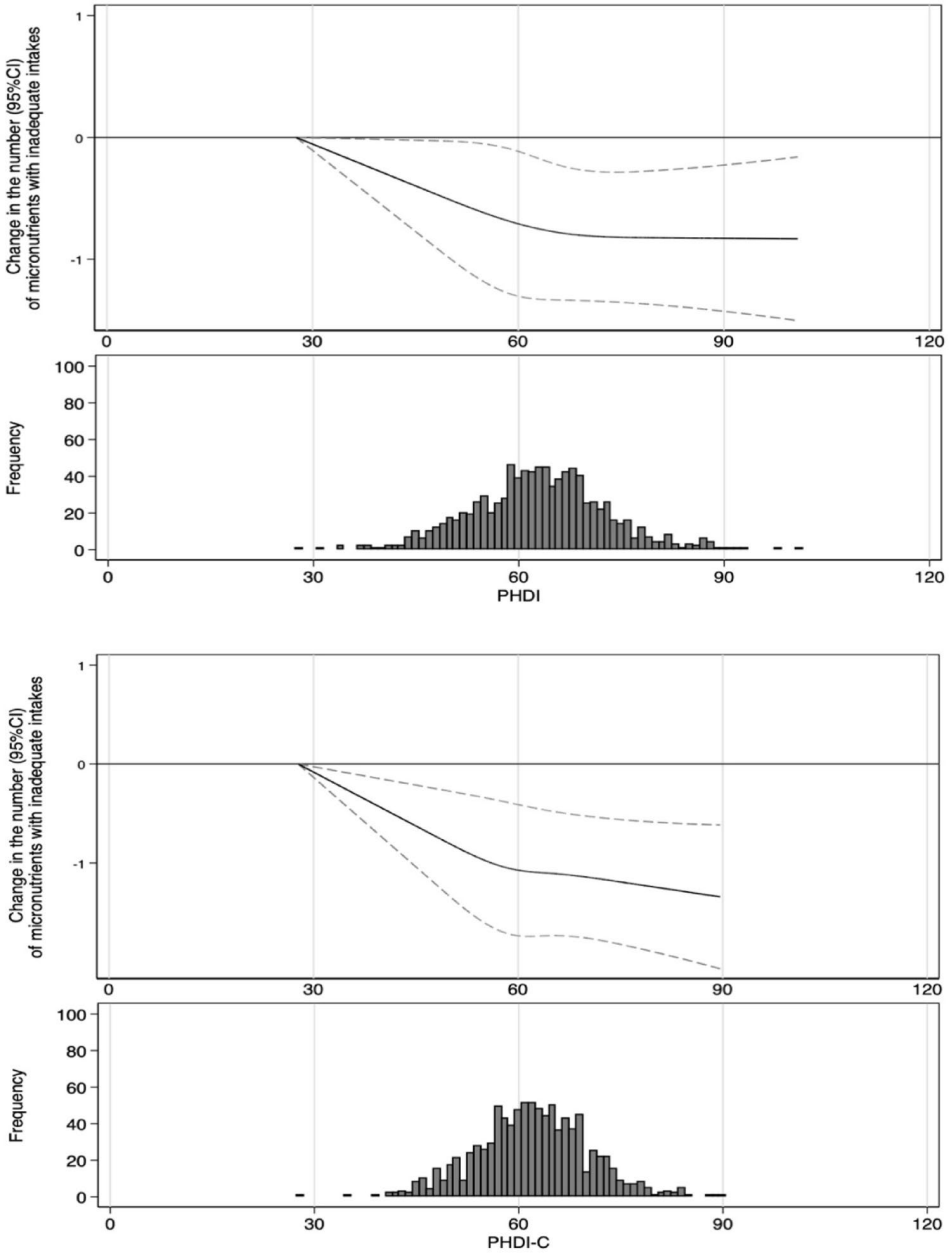
The splines above show the change (95% CI) in the number of micronutrient inadequacy associated with The PHDI and the PHDI-C. The histograms below show the frequency of participants by PHDI/PHDI-C. *Change* (y axis) refers to the predicted difference in the number of micronutrients with inadequate intakes associated with a given increase in the PHDI or PHDI-C score, estimated from the fitted regression model. Change (95% CI) in micronutrient inadequacies across PHDI and PHDI-C estimated by restricted cubic spline GEE models, adjusted for sex, age, energy intake, breastfeeding duration, parental knowledge about nutritional recommendations for children, parental attitudes towards child’s dietary habits, physical activity and screen time

This study included 945 participants (49.1% girls) with a mean age of 4.98 years (SD: 0.83). On average, 85.5% of the participants had at least one parent with higher education. Additionally, 12.17% of the participants were classified as overweight or obese based on BMI cut-off points. Overall adherence to the EAT Lancet diet was moderate, as measured by the PHDI, with a median score of 63.0 (IQR: 57.1–69.0) on a potential range of 0 to 150. PHDI Score varied between 27.6 and 100.8 (Fig. 1). Table 2 summarizes the participants’ main characteristics according to PHDI tertiles. Higher PHDI scores were observed in children who had longer breastfeed periods, were more physically active, spent less time in front of screens and who have parents with healthier attitudes towards their child’s dietary habits and greater knowledge of nutritional recommendations for their children. Similar associations were found when participants were classified according the PHDI-C. Descriptive characteristics and the range of scores for the PHDI-C are provided in Supplementary Table S1. The median score of the PHDI-C was 62.3 (IQR: 57.0–67.4) on a potential range of 0 to 150.

**Table 2:** Characteristics of participants and their families in the SENDO project by tertiles of the PHDI. Numbers are mean (SD) or N (%)

	T1	T2	T3	p for trend
Range of PHDI score	27.6–59.3	59.3–67.0	67.0–100.8	
n	315	315	315	
Sex (female), %	145 (46)	161 (51)	158 (50)	0.291
Age (years)	5.0 (± 0.8)	5.1 (± 0.9)	4.9 (± 0.8)	0.376
Race (white), %	301 (96)	300 (95)	301 (96)	0.996
Birthweight (g)	3252 (± 594.7)	3189 (± 511.0)	3275 (± 496.8)	0.662
Z-score of the BMI	0.00 (± 1.2)	0.11 (± 1.1)	0.00 (± 1.1)	0.909
Moderate-vigorous physical activity (h/day)^†^	1.04 (± 0.8)	1.09 (± 0.8)	1.26 (± 0.8)	< 0.001
Screen time (h/day)	1.22 (± 0.9)	1.06 (± 0.8)	1.00 (± 1.1)	0.003
Gestational age (weeks), %				0.499
< 38	46 (15)	43 (14)	41 (13)	
38 to 40	125 (40)	130 (131)	123 (134)	
> 40	142 (45)	141 (45)	149 (48)	
Birthweight (g), %				0.970
< 2500	34 (11)	27 (9)	20 (6)	
2500–3000	58 (18)	77 (25)	68 (21)	
3000–3500	119 (38)	131 (42)	130 (42)	
3500–4000	78 (25)	67 (21)	80 (26)	
> 4000	24 (8)	12 (4)	15 (5)	
Breastfeeding duration (months), %				< 0.001
No breastfeeding	68 (22)	49 (16)	33 (11)	
< 6	96 (30)	92 (29)	64 (20)	
6–12	78 (25)	87 (28)	72 (23)	
> 12	73 (23)	87 (28)	146 (46)	
Weight status, %				0.370
Low weight	50 (16)	45 (14)	54 (17)	
Normal weight	222 (71)	233 (74)	226 (72)	
Overweight/obesity	43 (14)	37 (12)	35 (11)	
Maternal age (years)	39.62 (± 4.7)	40.38 (± 4.0)	39.76 (± 4.1)	0.627
Parents higher education* %				
Both	151 (48)	181 (57)	182 (58)	0.082
At least one	257 (82)	270 (86)	281 (89)	0.088
Parental healthy attitudes towards child’s dietary habits, %				< 0.001
Low (< 40%)	19 (6)	19 (6)	7 (2)	
Moderate (40–70%)	132 (42)	99 (31)	70 (22)	
High (> 70%)	164 (52)	197 (63)	238 (76)	
Parental knowledge about the child’s nutritional recommendations, %				< 0.001
Low (< 40%)	86 (27)	59 (19)	60 (19)	
Moderate (40–70%)	203 (64)	208 (66)	201 (64)	
High (> 70%)	26 (8)	48 (15)	54 (17)	
Number of children, %				0.722
1	51 (16)	42 (13)	43 (14)	
2	171 (54)	150 (48)	190 (60)	
3–4	80 (25)	100 (32)	74 (24)	
5 or more	13 (4)	23 (7)	8 (3)	
Child’s position among siblings, %				0.527
The oldest/singletons	110 (35)	115 (37)	119 (38)	
2nd/3, 2nd or 3rd/4	39 (12)	58 (18)	35 (11)	
The youngest or beyond the 4th	166 (53)	142 (45)	161 (51)	

T1, tertile 1; T2, tertile 2; T3, tertile 3

*in posession of a university degree

^†^ annual average of hours/day spent doing moderate (≤ 5 METs/hour) or vigorous physical activity (> 5 METs/hour)

P for trend for continuous outcomes was obtained using generalized estimating equations, modeling the median value of each tertile of the PHDI/PHDI-C as a continuous variable. P for trend for categorical variables was derived from a chi-square test for linear trend

Higher PHDI scores were associated with higher total energy intake (TEI) (Table 3). Both indices were associated with a higher proportion of carbohydrates, and a lower proportion of protein. An inverse relation with the proportion of fat intake was observed for the PHDI-C score. Both indices were also linked to healthier fat profiles. As adherence to either score increased, cholesterol and saturated fat intake decreased, while monounsaturated and polyunsaturated fat intake increased. Moreover, higher scores in both indices were associated with greater intakes of linoleic acid, arachidonic acid, and linolenic acid. EPA and DHA also rise with higher scores, though this increase is only significant for the PHDI. Additionally, higher scores in both indices were associated with higher fiber intake.

**Table 3:** Dietary composition according to tertiles of PHDI and PHDI-C

	PHDI		PHDI-C	
	T1	T3	T1	T3
n	315	315	315	315
*Macronutrients*				
TEI (kcal)	1934 (481.7)	2050 (442.1)*	2024 (500.5)	2008 (444.0)
Carbohydrate intake (% of TEI)	43.20 (5.55)	44.30 (5.27)*	42.47 (5.46)	44.64 (5.07)*
Protein intake (% of TEI)	17.64 (2.18)	16.45 (1.96)*	17.38 (2.47)	16.75 (1.93)*
Fat intake (% of TEI)	39.15 (5.71)	39.26 (5.07)	40.15 (5.77)	38.61 (4.92)*
Cholesterol, mg/d	288.5 (85.30)	254.2 (64.48)*	288.31 (85.53)	258.06 (66.16)*
SFA intake (% of TEI)	11.61 (2.28)	10.38 (2.03)*	11.81 (2.34)	10.27 (1.96)*
MUFA intake (% of TEI)	14.45 (3.77)	15.89 (3.18)*	15.08 (4.00)	15.5 (3.08)
PUFA intake (% of TEI)	4.53 (1.22)	4.86 (1.13)*	4.45 (1.17)	4.82 (1.11)*
Linoleic cid (18:2n-6), g/d	8.11 (3.12)	9.10 (3.17)*	8.24 (3.02)	8.84 (3.00)*
Arachidonic acid (20:4n-6), g/d	0.11 (0.03)	0.10 (0.03)*	0.12 (0.04)	0.10 (0.03)*
Linolenic acid (18:3n-3), g/d	0.62 (0.19)	0.77 (0.25)*	0.64 (0.19)	0.75 (0.24)*
EPA (20:5n-3), mg/d	84.11 (48.91)	94.81 (49.32)*	88.80 (48.38)	89.54 (45.08)
DHA (22:6n-3), mg/d	152.7 (114.7)	183.7 (102.15)*	162.8 (114.8)	172.20 (103.9)
Fibre intake, g/d	17.56 (5.30)	25.22 (6.68)*	17.88 (5.25)	24.45 (6.78)*
*Food groups*^*†*^				
Vegetables	169.1 (111.4)	283.4 (120.7)*	171.9 (107.9)	270.5 (124.4)*
Fruits	310.2 (197.3)	499.1 (251.8)*	322.7 (201.8)	449.6 (231.9)*
Legumes	27.08 (15.90)	39.38 (22.96)*	26.61 (13.43)	39.48 (22.47)*
Cereal grains	81.11 (38.36)	105.2 (56.58)*	76.00 (41.53)	107.0 (52.67)*
Potatoes	23.93 (21.20)	35.31 (22.58)*	25.43 (25.29)	33.43 (19.96)*
Nuts	1.06 (0.98)	2.13 (1.43)*	1.14 (0.94)	2.10 (1.44)*
Dairy products	531.3 (243.5)	389.6 (227.7)*	598.1 (275.5)	397.0 (184.0)*
Eggs	20.63 (12.71)	20.19 (7.95)	19.20 (12.63)	21.11 (7.78)*
Fish and Seafood	32.40 (17.75)	36.37 (15.43)*	34.25 (17.3)	34.82 (15.42)
Meat	137.4 (44.33)	120.4 (45.8)*	135.6 (43.91)	119.85 (43.48)*
Fastfood	59.24 (28.53)	57.81 (29.85)	58.46 (28.09)	56.34 (28.30)
Pastries and sweets	87.23 (62.55)	82.50 (54.11)*	96.45 (71.59)	63.09 (40.11)*
Sugar-sweetened beverages	45.88 (74.38)	31.14 (52.25)*	56.98 (96.70)	31.39 (44.36)*
Olive oil	14.888 (13.28)	21.09 (12.19)*	18.14 (15.89)	19.60 (10.75)
Other vegetable oils	2.67 (4.00)	2.52 (3.68)	2.51 (3.79)	2.55 (3.85)

Mean (SD)

TEI, Total energy intake; SFA, Saturated fatty acids; MUFA, Monounsaturated fatty acids; PUFA, Polyunsaturated fatty acids; T1, Tertile 1; T3, Tertile 3

*p for trend < 0.05, ^**†**^ grams/day

P for trend for continuous outcomes was obtained using generalized estimating equations, modeling the median value of each tertile of the PHDI/PHDI-C as a continuous variable

Children with higher scores in both indices consumed more vegetables, fruits, legumes, cereal grains, potatoes, and nuts, and fewer dairy products, pastries and sweets, sugar-sweetened beverages and meat (Table 3). Children with higher PHDI-C scores also consumed more eggs, and those with higher PHDI scores had higher intakes of olive oil.

We calculated the mean intake for each micronutrient by tertile of PHDI and PHDI-C (Table 4) and the energy-adjusted increase in micronutrient intake for each 10-point increase of the PHDI and PHDI-C scores (Table S3). Participants with higher PHDI and PHDI-C scores had higher intakes of vitamins A, C, E, B1, B3, and B6, as well as folate, Fe, Mg, Zn, Cr, K, and Se (only for the PHDI-C). However, higher adherence to the EAT-Lancet diet was associated with lower intakes of vitamins B2 and B12, and Ca, and I (Table 3 and S3).

**Table 4:** Absolute intake of micronutrients by tertiles of PHDI and PHDI-C

	PHDI		PHDI-C	
	T1	T3	T1	T3
n	315	315	315	315
*Micronutrients*				
Vitamin A (RE) (µg/d)	972.6 (542.4)	1156 (419.4)*	962.8 (487.7)	1159 (450.9)*
Vitamin C (mg/d)	109.8 (52.0)	178.2 (70.94)*	114.6 (55.07)	165.5 (70.89)*
Vitamin D (µg/d)	2.89 (1.85)	3.24 (1.63) *	3.02 (1.82)	3.13 (1.67)
Vitamin E (mg/d)	7.19 (3.21)	9.55 (3.11)*	7.67 (3.42)	9.10 (3.13)*
Vitamin B1 (mg/d)	1.31 (0.34)	1.52 (0.34)*	1.34 (0.34)	1.51 (0.34)*
Vitamin B2 (mg/d)	1.97 (0.63)	2.01 (0.60)	2.09 (0.68)	1.98 (0.55)*
Vitamin B3 (mg/d)	33.60 (9.02)	36.87 (9.24)*	34.32 (9.19)	36.33 (9.34)*
Vitamin B6 (mg/d)	2.08 (0.54)	2.52 (0.55)*	2.13 (0.54)	2.46 (0.57)*
Folate (µg/d)	261.6 (85.86)	339.9 (92.35)*	269.2 (87.97)	330.9 (92.41)*
Vitamin B12 (µg/d)	4.68 (1.59)	4.43 (1.42)*	4.73 (1.53)	4.43 (1.47)*
Ca (mg/d)	1149 (362.5)	1114 (321.2)	1236 (396.8)	1102 (302.0)*
I (µg/d)	108.2 (31.05)	103.9 (29.65)	114.6 (33.35)	103.5 (26.91)*
Fe (mg/d)	12.62 (3.08)	15.17 (3.14)*	12.75 (3.02)	14.87 (3.19)*
P (mg/d)	1584 (657.4)	1769.9 (734.6)*	1706 (719.1)	1776 (747.1)
Mg (mg/d)	272.3 (65.18)	325.0 (70.25)*	282.5 (67.57)	319.0 (71.62)*
Se (µg/d)	69.22 (17.68)	72.02 (17.69)	68.77 (17.45)	72.76 (18.64)*
Zn (mg/d)	9.06 (2.64)	9.90 (2.64)*	9.40 (2.78)	9.60 (2.53)
Cr (µg/d)	60.08 (22.95)	70.23 (22.89)*	63.99 (26.71)	68.33 (22.98)*
K (mg/d)	3084 (860.4)	3701 (882.8)*	3228.64 (893.0)	3591 (885.8)*
Na (mg/d)	2811 (1002)	2935 (1064)	2895.83 (1011)	2860 (1047)

Mean (SD)

RE, Retinol equivalents, T1, tertile 1; T3, tertile 3

*p for trend < 0.05

*P* for trend for continuous outcomes was obtained using generalized estimating equations, modeling the median value of each tertile of the PHDI/PHDI-C as a continuous variable

We calculated the prevalence of inadequacy for each micronutrient according to tertiles of PHDI and PHDI-C (Table S3). Participants in the highest tertile of both indices had a lower prevalence of inadequacy of vitamin E. Participants in T3 of PHDI-C had lower point estimates for prevalence of inadequacy for Ca and I than those in T3 of the PHDI.

The splines in Fig. 1 illustrate the relationship between PHDI and PHDI-C scores and the number of micronutrients with inadequate intake (solid line) with 95% CI (dashed line). After adjusting for potential confounders, we observed that the number of inadequate micronutrient intakes decreased as the PHDI-C score improved, displaying an inverse linear association (*p* < 0.001). However, this pattern was not observed for the PHDI, where the initial reduction in the number of inadequate intakes plateaued at scores above 70.

Table 5 presents the OR and 95% CI for unmet EARs for ≥ 3 micronutrients across PHDI and PHDI-C tertiles. We found a significant linear trend in the odds of having ≥ 3 micronutrient inadequacies across tertiles of both PHDI and PHDI-C. Compared to T1, children in T3 of the PHDI and PHDI-C had a 64% and 57% lower odds of having ≥ 3 micronutrient inadequacies in the crude model, respectively. In the fully adjusted model, children in T3 of PHDI and PHDI-C had a 30% and 55% lower odds of having ≥ 3 micronutrient inadequacies, and the linear trend was only significant for the PHDI-C (*p* = 0.025).

**Table 5:** OR and 95%CI for inadequate intake of 3 or more micronutrients associated with tertiles of PHDI and PHDI-C

	OR (95% CI)			
PHDI	T1	T2	T3	p for trend
% of participants with ≥ 3 inadequate intakes of micronutrients	16.51	4.44	6.67	
Crude	1.00 (ref)	0.24 (0.13–0.44)	0.36 (0.21–0.61)	< 0.001
Multivariate adjusted model 1	1.00 (ref)	0.39 (0.20–0.76)	0.53 (0.27–1.02)	0.038
Multivariate adjusted model 2	1.00 (ref)	0.40 (0.20–0.77)	0.62 (0.29–1.35)	0.131
Multivariate adjusted model 3	1.00 (ref)	0.43 (0.32–1.54)	0.70 (0.32–1.54)	0.235

	OR (95% CI)			
PHDI-C	T1	T2	T3	p for trend
% of participants with ≥ 3 inadequate intakes of micronutrients	14.29	6.67	6.67	
Crude	1.00 (ref)	0.43 (0.25–0.75)	0.43 (0.25–0.74)	0.002
Multivariate adjusted model 1	1.00 (ref)	0.41 (0.21–0.80)	0.39 (0.21–0.75)	0.004
Multivariate adjusted model 2	1.00 (ref)	0.47 (0.23–0.93)	0.43 (0.21–0.88)	0.016
Multivariate adjusted model 3	1.00 (ref)	0.49 (0.25–0.98)	0.45 (0.22–0.94)	0.025

Mean (SD)

Estimates derived from generalized estimating equations with an identity link and exchangeable correlation structure (Gaussian family), using maternal ID as the clustering variable. The exposure was tertiles of adherence to the PHDI/PHDI-C, and the outcome was the probability of having ≥ 3 inadequate nutrient intakes

Model 1: adjusted for sex (male vs. female), age (continuous), and energy intake (continuous);

Model 2: additionally adjusted for breastfeeding duration (none, < 6, 6–12, and > 12 months), parental knowledge about the child’s nutritional recommendations (low, medium, and high score) for children, and parental attitudes towards child’s dietary habits (low, medium, and high score);

Model 3: additionally adjusted for moderate–vigorous physical activity (continuous) and screen time (continuous)

**Fig. 2:**
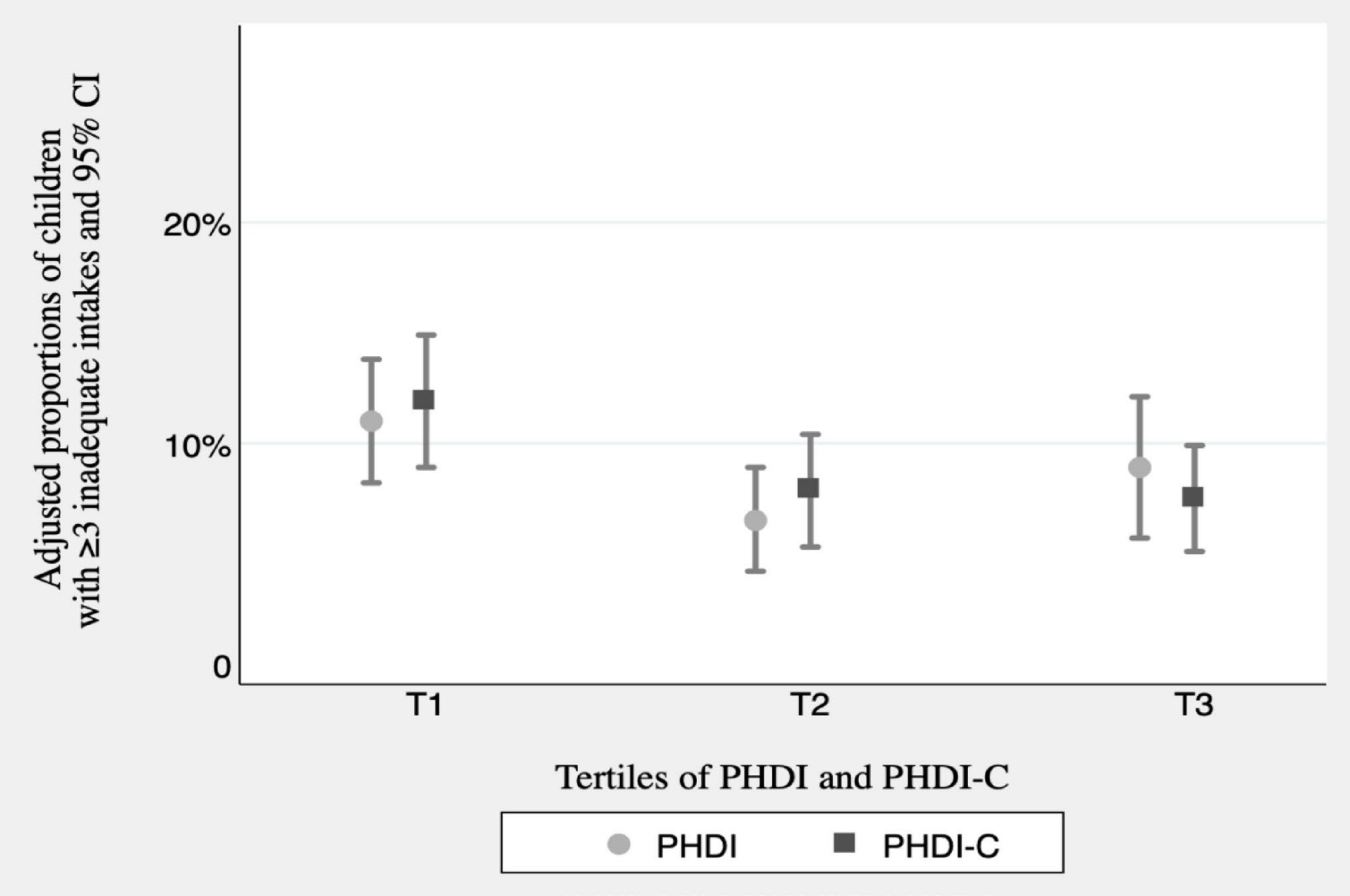
Adjusted proportions of children with ≥ 3 inadequate micronutrient intake (95% CI) in each tertile of the PHDI and PHDI-C Scores. Estimates derived from generalized estimating equations with an identity link and exchangeable correlation structure (Gaussian family), using maternal ID as the clustering variable. The exposure was tertiles of adherence to the PHDI/PHDI-C, and the outcome was the probability of having ≥ 3 inadequate nutrient intakes. Adjusted for sex, age, energy intake, breastfeeding duration, parental knowledge about nutritional recommendations for children, parental attitudes towards child’s dietary habits, physical activity and screen time

To further explore the relationship between dietary adherence and micronutrient adequacy, we calculated the marginal effect of PHDI and PHDI-C on the risk of having an inadequate intake of ≥ 3 micronutrients, this is, the multivariate-adjusted proportion of children with inadequate intake of ≥ 3 micronutrient in each tertile of both indices (Fig. 2). After accounting for all potential confounders, the adjusted proportions of children with ≥ 3 micronutrient inadequacies of the PHDI-C were 11.9% (95% CI: 8.9–14.9%), 7.9% (95% CI; 5.3–10.4%), 7.5% (95% CI: 5.1.–10.0%), in T1, T2 and T3 respectively. Regarding the PHDI, the adjusted proportions were 11.0% (95% CI: 8.3–13.8%), 6.5% (95% CI; 4.2–8.9%), 8.9% (95% CI: 5.7–12.1%), in T1, T2 and T3 respectively. These results suggest that better adherence to the PHDI-C shows a stronger association with improved nutrient adequacy than adherence to the PHDI.

In our sensitivity analysis using 1400 kcal as a reference intake for this age range and calculating micronutrient intakes proportionally, we found that micronutrient intakes were lower overall (Table S4). However, overall similar trends in micronutrient adequacy were observed (Table S5). Notably, vitamins A and C and folate showed lower prevalence of inadequacy in T3 of both indices, and Ca and I showed higher prevalence of inadequacy.

## Discussion

The present cross-sectional study explored whether the PHDI-C, an adaptation of the PHDI for children and adolescents, is associated with nutrient adequacy and compared its performance with the original version of the PHDI. The PHDI-C introduces changes to the PHDI, modifying certain intake values of the different food groups, therefore indirectly testing whether slight adjustments to the EAT-Lancet recommendations in children might better accommodate growing children’s nutritional needs. To the best of our knowledge, this is the first study assessing micronutrient adequacy of the EAT-Lancet diet in young children in a real-world setting. Moreover, no previous study has directly compared the PHDI-C with the PHDI in terms of nutrient adequacy, to assess if the proposed changes are in fact a better fit for children’s dietary needs. Our results indicate that higher PHDI-C scores were associated with fewer unmet EARs than the original PHDI score.

As expected, both indices were positively associated with higher intakes of nutrients predominantly derived from plant-source foods, some of which are considered nutrients of public health concern, such as fiber, K, and Fe [31]. These findings are consistent with previous studies in adult population [13, 32]. In contrast, both indices were inversely associated with nutrients primarily obtained from animal- source foods, including vitamins B2 and B12 and I and Ca, which are commonly lacking in diets worldwide [17, 33]. These results align with those obtained in the original validation study of the PHDI conducted in adults [19].

With regard to micronutrient adequacy, higher scores in both indices were associated with a lower prevalence of vitamin E inadequacy. However, for Ca and I—the micronutrients with the highest rates of inadequacy in our participants—higher scores in either index were not associated with increased adequacy. Participants in the highest tertile of the PHDI-C did show lower point estimates of Ca and I inadequacy compared with those in the highest tertile of the PHDI. Our sensitivity analysis based on a 1400 kcal reference diet further showed that higher adherence to the PHDI and PHDI-C was significantly associated with (*p for trend* < *0.01*) higher prevalence of inadequacy of Ca and I.

Previous research provides complementary insights. A study conducted in Norway that assessed the environmental and nutritional consequences of shifting 2-year-old children’s diets towards a theoretical EAT-Lancet scenario diet found that this transition improved micronutrient adequacy, except for Se, Ca and I [34]. Similarly, a study by Lassen et al. using Danish food composition data showed that the EAT-Lancet diet would be deficient in vitamins A and D, Ca, I and Se for the population aged 6–65 years [30]. In contrast, research conducted in European adolescents found that Ca and I intake increased with higher PHDI scores [12].

Despite the higher thresholds for animal-based food intake in the PHDI-C, greater adherence to this index did not significantly improve I or Ca adequacy, even though dairy and eggs are recognized sources of these micronutrients [35, 36]. This raises the question of whether the dietary targets proposed by the EAT-Lancet diet—even when slightly adapted in the PHDI-C—remain too restrictive to meet children’s Ca and I requirements.

In the recently updated report, the EAT-Lancet Commission points out that the intake of Ca, vitamin B12, Fe, and I within the PHD warrants further attention, particularly in populations with low dietary diversity. However, the authors consider that optimization within the PHD reference values for general food groups (e.g., increasing the proportion of green leafy vegetables for Fe, fermented soy foods for vitamin B12, and algae for vitamin B12 and I) can ensure nutritional adequacy across all population groups [37].

### Strengths and limitations

We acknowledge that our study has both strengths and limitations. To the best of our knowledge, this is the first study to analyze the relationship between adherence to the EAT-Lancet diet and nutrient adequacy in young children, using two novel dietary indices. It also provides initial insights into the performance of the PHDI-C, which introduces modifications to the EAT-Lancet diet to better accommodate the nutritional needs of children, addressing the limitations pointed out in previous studies [15, 38]. Another strength is the use of Generalized Estimating Equation models, which account for the potential correlation between siblings—a common challenge in pediatric studies.

Despite these contributions, several limitations must be acknowledged. First, our study population exhibited moderate adherence to the PHD, a trend similar to findings in other studies [12, 20, 39]. Even among participants in the highest tertile, animal-derived product consumption remained well above the dietary targets set by the EAT-Lancet diet [3], though lower than levels reported in population-based studies in Spain [40]. This aspect is relevant because our study may not fully capture the impact of strict adherence to the EAT-Lancet dietary pattern. It is possible that higher adherence could enhance nutrient adequacy, while severely restricted intakes of animal-source foods could also exacerbate potential nutrient shortfalls. Nevertheless, despite a large proportion of participants clustering around scores of approximately 50–70—which encompassed the entire middle tertile—we were still able to detect significant linear trends across tertiles. This suggests that the observed associations are robust and not driven by extreme values, a finding further supported by our supplemental analysis using a 10-point increase in score. Future research in populations with higher adherence to the EAT-Lancet diet may provide deeper insight into this balance.

Second, our participants primarily belonged to families with higher socioeconomic status and highly educated parents. This factor is important because higher parental education is often associated with better diet quality. In fact, our participants tended to have better diet quality than that of the average Spanish child [41, 42]. Furthermore, individuals enrolled in nutrition-focused cohort studies tend to be more health-conscious, potentially leading to fewer observed nutrient inadequacies compared to population-based studies [43]. Although a larger, more socioeconomically diverse sample might provide broader insights, this limitation does not invalidate our conclusions. Rather, our results should be interpreted in the context of underlying mechanisms rather than the sample’s representativeness [34]. Additionally, the high level of parental education may be advantageous, as it likely improves the validity of self-reported dietary data and reduces potential confounding factors related to socioeconomic disparities [44, 45]. Third, another limitation relates to self-reported dietary data, which may introduce measurement errors. FFQs tend to overestimate food intake, potentially leading to an underestimation of micronutrient inadequacies. However, the FFQ used in this study has been previously validated in our population [22], and we excluded participants with extreme energy and micronutrient intakes to minimize bias. We also performed a sensitivity analysis, similar to that of Lassen and colleagues [30], using 1400 kcal as a reference intake for this age range and calculating micronutrient intakes proportionally. Nonetheless, the primary aim of the study was not to evaluate absolute intake levels but rather to assess linear trends in micronutrient adequacy according to adherence to both indices, and these trends remained robust in the sensitivity analysis. Fourth, we assessed the probability of micronutrient adequacy rather than actual micronutrient deficiencies, which would require biomarker analysis for a more precise evaluation. Fifth, absolute intakes of micronutrients may have been underestimated because we did not consider the intake of foods fortified with vitamins, minerals, or supplements that participants may be taking. Finally, as this is an observational study, we cannot completely rule out residual confounding from unmeasured factors. However, the comprehensive questionnaire used allowed us to adjust for multiple potential confounders, strengthening the reliability of our findings.

## Conclusion

This study provides novel insights into the relationship between adherence to the PHD and micronutrient adequacy in young children, comparing the performance of the PHDI and its child-adapted version, the PHDI-C. Our findings suggest that higher adherence is associated with improved micronutrient adequacy, with a slightly stronger association with nutritional adequacy for the PHDI-C as compared to the original PHDI. Future research should further investigate the impact of the EAT-Lancet diet in populations with higher adherence and assess the PHDI-C’s validity in measuring the environmental sustainability of diets in children and adolescents in relation to the EAT-Lancet targets.

## Supplementary Information

Supplementary Material 1

## Data Availability

The datasets used and/or analysed during the current study are available from the corresponding author on reasonable request.

## Abbreviations

AI: Adequate intake
BMI: Body mass index
CI: Confidence interval
DHA: Docosahexaenoic acid
EAR: Estimated average requirement
EPA: Eicosapentaenoic acid
FFQ: Food-frequency questionnaire
PHD: Planetary health diet
PHDI: Planetary health diet index
PHDI-C: Planetary health diet index adapted to children and adolescents
OR: Odds ratio
SD: Standard deviation
SENDO: Seguimiento del Niño para un Desarrollo Óptimo
T1: Tertile 1
T2: Tertile 2
T3: Tertile 3
TEI: Total energy intake
Ca: Calcium
I: Iodine
Fe: Iron
P: Phosphorous
M: Magnesium
Se: Selenium
Zn: Zinc
Cr: Chromium
K: Potassium
Na: Sodium
