# Supplementary material for: EAT-Lancet reference diet and nutritional adequacy in children: examining the planetary health diet index for children (PHDI-C)
Source: Eur J Nutr. 2025 Dec 19;65(1):16. doi: 10.1007/s00394-025-03858-9 (PMC12717126; doi:10.1007/s00394-025-03858-9)
Supplement: Supplementary file 1 — Supplementary Material 1 [file 394_2025_3858_MOESM1_ESM.docx]

**SUPPLEMENTARY MATERIAL**

**Table S1.** Characteristics of participants and their families in the SENDO project by tertiles of the PHDI-C. Numbers are mean (sd) or N (%)

|  | **T1** | **T2** | **T3** | p for trend |
| --- | --- | --- | --- | --- |
| **Range of PHDI-C score** | 27.8-58.9 | 58.9-65.4 | 65.4-89.6 |  |
| **n** | 315 | 315 | 315 |  |
|  |  |  |  |  |
| **Sex (female), %** | 143 (45) | 168 (53) | 153 (49) | 0.391 |
| **Age (years)** | 5.1 (±0.9) | 5.0 (0.8) | 4.9 (0.7) | 0.002 |
| **Race (white), %** | 303 (96) | 296 (94) | 303 (96) | 0.959 |
| **Birthweight (g)** | 3269 (±581.2) | 3195 (±518.4) | 3251 (±506.1) | 0.669 |
| **Z-score of the BMI** | 0.1 (±1.1) | 0.1 (±1.2) | -0.1 (±1.1) | 0.048 |
| **Moderate-vigorous physical activity (h/day) ^†^** | 1.1 (±0.8) | 1.2 (0.8) | 1.2 (0.8) | 0.039 |
| **Screen time (h/day)** | 1.2 (±0.9) | 1.1 (±1.1) | 1.0 (±0.7) | <0.001 |
| **Gestational age (weeks), %** |  |  |  | 0.616 |
| <38 | 46 (15) | 42 (14) | 42 (13) |  |
| 38 to 40 | 114 (36) | 136 (44) | 128 (41) |  |
| >40 | 153 (49) | 134 (43) | 145 (46) |  |
| **Birthweight (g), %** |  |  |  | 0.457 |
| <2500 | 29 (9) | 33 (11) | 19 (6) |  |
| 2500-3000 | 58 (19) | 67 (21) | 78 (25) |  |
| 3000-3500 | 129 (38) | 138 (44) | 122 (39) |  |
| 3500-4000 | 81 (26) | 78 (25) | 78 (25) |  |
| >4000 | 22 (7) | 14 (5) | 15 (5) |  |
| **Breastfeeding duration (months), %** |  |  |  | <0.001 |
| No breastfeeding | 62 (20) | 53 (17) | 35 (11) |  |
| <6 | 94 (30) | 91 (29) | 67 (21) |  |
| 6 to 12 | 81 (26) | 78 (25) | 78 (25) |  |
| >12 | 78 (25) | 93 (30) | 135 (43) |  |
| **Weight Status (%)** |  |  |  | 0.019 |
| Low weight | 39 (12) | 53 (17) | 57 (18) |  |
| Normal weight | 232 (74) | 222 (71) | 227 (72) |  |
| Overweight/obesity | 44 (14) | 40 (13) | 31 (10) |  |
|  |  |  |  |  |
| **Maternal age (years)** | 40.29 (±4.3) | 39.75 (±4.5) | 39.72 (±4.0) | 0.092 |
| **Parents higher education*** **%** |  |  |  |  |
| Both | 158 (50) | 138 (44) | 135 (43) | 0.062 |
| At least one | 260 (83) | 274 (87) | 274 (87) | 0.107 |
| **Parental healthy attitudes towards child’s dietary habits, %** |  |  |  | <0.001 |
| Low (<40%) | 17 (5) | 18 (6) | 10 (3) |  |
| Moderate (40-70%) | 131 (42) | 95 (30) | 75 (24) |  |
| High (>70%) | 167 (53) | 202 (64) | 230 (73) |  |
| **Parental knowledge about the child’s nutritional recommendations, %** |  |  |  | 0.001 |
| Low (<40%) | 84 (27) | 62 (20) | 59 (19) |  |
| Moderate (40-70%) | 199 (63) | 212 (67) | 201 (64) |  |
| High (>70%) | 32 (10) | 41 (13) | 55 (18) |  |
| **Number of children, %** |  |  |  | 0.03 |
| 1 | 46 (14) | 46 (15) | 44 (14) |  |
| 2 | 164 (52) | 157 (50) | 190 (60) |  |
| 3-4 | 85 (27) | 98 (31) | 71 (22) |  |
| 5 or more | 20 (6) | 14 (4) | 10 (3) |  |
| **Child’s position among siblings, %** |  |  |  | 0.987 |
| The oldest/singletons | 110 (35) | 116 (37) | 118 (38) |  |
| 2^nd^/3, 2^nd^ or 3^rd^/4 | 50 (16) | 48 (15) | 34 (11) |  |
| The youngest or beyond the 4^th^ | 155 (49) | 151 (48) | 163 (52) |  |
|  |  |  |  |  |
|  |  |  |  |  |

*in posession of a university degree

**^†^** annual average of hours/day spent doing moderate (≤ 5 METs/hour) or vigorous physical activity (>5 METs/hour)

P for trend for continuous outcomes was obtained using generalized estimating equations, modeling the median value of each tertile of the PHDI/PHDI-C as a continuous variable. P for trend for categorical variables was derived from a chi-square test for linear trend.

**Table S2.** Prevalence (%) of inadequate micronutrient intake according to tertiles of PHDI and PHDI-C

|  | PHDI | | PHDI-C | |  |
| --- | --- | --- | --- | --- | --- |
|  | T1 | T3 | T1 | T3 | |
| n | 315 | 315 | 315 | 315 | |
|  |  |  |  |  | |
| **N,% Inadequate Intake** |  |  |  |  | |
| **Vitamin A (equiv Retinol)** | 3 (1.0) | 0 (0.0) | 1 (0.3) | 1 (0.3) | |
| **Vitamin C** | 4 (1.3) | 0 (0.0)* | 3 (1.0) | 0 (0.0) | |
| **Vitamin D** | 315 (100.0) | 315 (100.0) | 315 (100.0) | 315 (100.0) | |
| **Vitamin E** | 132 (41.9) | 42 (13.3)* | 109 (34.6) | 52 (16.5)* | |
| **Vitamin B1** | 0 (0.0) | 0 (0.0) | 0 (0.0) | 0 (0.0) | |
| **Vitamin B2** | 0 (0.0) | 0 (0.0) | 0 (0.0) | 0 (0.0) | |
| **Vitamin B3** | 0 (0.0) | 0 (0.0) | 0 (0.0) | 0 (0.0) | |
| **Vitamin B6** | 0 (0.0) | 0 (0.0) | 0 (0.0) | 0 (0.0) | |
| **Folate** | 5 (1.6) | 0 (0.0) | 3 (1.0) | 1 (0.3) | |
| **Vitamin B12** | 0 (0.0) | 1 (0.3) | 0 (0.0) | 1 (0.3) | |
| **Ca** | 22 (7.0) | 18 (5.7) | 19 (6.0) | 14 (4.4) | |
| **I** | 14 (4.4) | 26 (8.3) | 16 (5.1) | 18 (5.7) | |
| **Fe** | 0 (0.0) | 0 (0.0) | 0 (0.0) | 0 (0.0) | |
| **P** | 0 (0.0) | 0 (0.0) | 0 (0.0) | 0 (0.0) | |
| **Mg** | 0 (0.0) | 0 (0.0) | 0 (0.0) | 0 (0.0) | |
| **Se** | 0 (0.0) | 0 (0.0) | 0 (0.0) | 0 (0.0) | |
| **Zn** | 0 (0.0) | 0 (0.0) | 2 (0.6) | 0 (0.0) | |
| **Cr** | 1 (0.3) | 0 (0.0) | 0 (0.0) | 0 (0.0) | |

*Prevalence of inadequacy has not been calculated for K and Na given that an EAR is lacking for both these micronutrients. Median intakes in all groups of both indices exceed the AI for K and Na, suggesting that risk of inadequate intake is low.*

*p for trend < 0.05

Estimates derived from generalized estimating equations with an identity link and exchangeable correlation structure (Gaussian family), using maternal ID as the clustering variable. The exposure was tertiles of adherence to the PHDI or PHDI-C, and the outcome was inadequate intake of each of the micronutrients listed above.

**Table S3.** Energy-adjusted increase in micronutrient intake for each 10-point increase of the PHDI and PHDI-C scores

|  | **PHDI** | | | **PHDI-C** | | |
| --- | --- | --- | --- | --- | --- | --- |
|  | **Beta coefficient** | **CI** | **p values** | **Beta coefficient** | **CI** | **p values** |
|  |  |  |  |  |  |  |
| **Micronutrients** |  |  |  |  |  |  |
| Vitamin A (equiv Retinol) (µg/d) | 82.92 | (53.92 to 111.91) | *p*<0.01 | 106.0 | (71.78 to 140.03) | *p*<0.01 |
| Vitamin C (mg/d) | 31.08 | (27.42 to 34.74) | *p*<0.01 | 30.67 | (26.14 to 35.20) | *p*<0.01 |
| Vitamin D (µg/d) | 0.07 | (-0.04 to 0.19) | 0.204 | 0.05 | (-0.08 to 0.19) | 0.489 |
| Vitamin E (mg/d) | 0.94 | (0.77 to 1.11) | *p*<0.01 | 0.86 | (0.65 to 1.07) | *p*<0.01 |
| Vitamin B1 (mg/d) | 0.07 | (0.06 to 0.08) | *p*<0.01 | 0.10 | (0.09 to 0.12) | *p*<0.01 |
| Vitamin B2 (mg/d) | -0.04 | (-0.07 to -0.01) | *p*<0.01 | -0.04 | (-0.08 to -0.01) | 0.020 |
| Vitamin B3 (mg/d) | 0.77 | (0.27 to 1.28) | *p*<0.01 | 1.44 | (0.85 to 2.04) | *p*<0.01 |
| Vitamin B6 (mg/d) | 0.17 | (0.14 to 0.20) | *p*<0.01 | 0.20 | (0.17 to 0.24) | *p*<0.01 |
| Folate (µg/d) | 34.49 | (29.89 to 39.08) | *p*<0.01 | 37.58 | (32.03 to 43.12) | *p*<0.01 |
| Vitamin B12 (µg/d) | -0.21 | (-0.30 to -0.13) | 0.012 | -0.10 | (-0.20 to 0.00) | *p*<0.01 |
| Ca (mg/d) | -50.07 | (-66.12 to -34.03) | *p*<0.01 | -68.47 | (-87.36 to -49.58) | *p*<0.01 |
| I (µg/d) | -4.59 | (-6.04 to -3.14) | *p*<0.01 | -4.67 | (-6.40 to -2.94) | *p*<0.01 |
| Fe (mg/d) | 0.97 | (0.85 to 1.10) | *p*<0.01 | 1.27 | (1.12 to 1.42) | *p*<0.01 |
| P (mg/d) | 43.96 | (1.84 to 86.07) | 0.041 | 45.83 | (-4.10 to 95.76) | 0.071 |
| Mg (mg/d) | 21.11 | (18.37 to 23.84) | *p*<0.01 | 24.72 | (21.47 to 27.97) | *p*<0.01 |
| Se (µg/d) | -0.01 | (-0.86 to 0.85) | 0.989 | 2.50 | (1.50 to 3.50) | *p*<0.01 |
| Zn (mg/d) | 0.17 | (0.04 to 0.30) | 0.011 | 0.22 | (0.06 to 0.37) | *p*<0.01 |
| Cr (µg/d) | 3.37 | (2.07 to 4.67) | *p*<0.01 | 3.33 | (1.78 to 4.87) | *p*<0.01 |
| K (mg/d) | 237.3 | (198.8 to 275.8) | *p*<0.01 | 250.2 | (203.9 to 296.6) | *p*<0.01 |
| Na (mg/d) | -28.90 | (-85.22 to 27.41) | 0.314 | 8.27 | (-58.50 to 75.03) | 0.801 |

Energy-adjusted differences in micronutrient intake (per 10-point increase in PHDI or PHDI-C) estimated using simple linear regression models with the continuous score as the independent variable.

**Table S4.** Sensitivity Analysis: Proportional micronutrients intake by tertiles of PHDI and PHDI-C based on a 1400 kcal reference diet. Mean (sd)

|  | **PHDI** | | **PHDI-C** | |
| --- | --- | --- | --- | --- |
|  | T1 | T3 | T1 | T3 |
| n | 315 | 315 | 315 | 315 |
| **Micronutrients** |  |  |  |  |
| Vitamin A (RE) (µg/d) | 710.62 (371.85) | 798.63 (271.04)* | 673.67 (329.11) | 816.40 (293.90)* |
| Vitamin C (mg/d) | 80.68 (36.60) | 123.79 (48.78)* | 79.96 (35.62) | 116.79 (48.41)* |
| Vitamin D (µg/d) | 2.15 (1.41) | 2.25 (1.10) | 2.15 (1.33) | 2.20 (1.13) |
| Vitamin E (mg/d) | 5.22 (2.00) | 6.56 (1.82)* | 5.31 (2.06) | 6.37 (1.85)* |
| Vitamin B1 (mg/d) | 0.96 (0.17) | 1.05 (0.17)* | 0.94 (0.17) | 1.06 (0.16)* |
| Vitamin B2 (mg/d) | 1.44 (0.34) | 1.38 (0.35)* | 1.46 (0.37) | 1.39 (0.31)* |
| Vitamin B3 (mg/d) | 24.90 (6.19) | 25.44 (4.99) | 24.30 (6.10) | 25.58 (5.04)* |
| Vitamin B6 (mg/d) | 1.54 (0.35) | 1.75 (0.35)* | 1.51 (0.33) | 1.74 (0.35)* |
| Folate (µg/d) | 191.42 (51.17) | 235.73 (58.76)* | 187.81 (48.60) | 233.36 (55.77)* |
| Vitamin B12 (µg/d) | 3.46 (1.11) | 3.05 (0.83)* | 3.34 (1.02) | 3.11 (0.89)* |
| Ca (mg/d) | 835.10 (182.73) | 764.82 (165.16)* | 858.70 (203.70) | 770.01 (136.19)* |
| I (µg/d) | 79.13 (16.34) | 71.39 (15.41)* | 80.20 (18.08) | 72.48 (12.38)* |
| Fe (mg/d) | 9.26 (1.51) | 10.49 (1.66)* | 8.93 (1.35) | 10.48 (1.56)* |
| P (mg/d) | 1154.03 (390.99) | 1222.98 (511.77) | 1189.31 (442.41) | 1245.44 (490.59) |
| Mg (mg/d) | 199.39 (27.80) | 225.01 (39.39)* | 197.52 (27.29) | 225.01 (38.00)* |
| Se (µg/d) | 51.01 (9.86) | 49.58 (8.58)* | 48.46 (9.59) | 51.07 (8.74)* |
| Zn (mg/d) | 6.63 (1.41) | 6.84 (1.58) | 6.57 (1.46) | 6.78 (1.51) |
| Cr (µg/d) | 43.97 (14.90) | 48.09 (12.35)* | 44.37 (15.83) | 47.65 (12.32)* |
| K (mg/d) | 2254.33 (424.10) | 2552.71 (471.92)* | 2254.45 (419.71) | 2525.02 (467.41)* |
| Na (mg/d) | 2054.79 (648.60) | 2006.38 (632.36) | 2021.30 (599.81) | 1993.67 (608.36) |

RE, Retinol Equivalents

*p for trend < 0.05

P for trend obtained from linear regression of the continuous variable across tertiles of PHDI/PHDI-C, assigning the median value of each tertile as a continuous variable.

**Table S5.** Sensitivity Analysis: Prevalence (%) of inadequate micronutrient intake according to tertiles of PHDI and PHDI-C based on a 1400 kcal reference diet. Mean (sd)

|  | PHDI | | PHDI-C | |  |
| --- | --- | --- | --- | --- | --- |
|  | T1 | T3 | T1 | T3 | |
| n | 315 | 315 | 315 | 315 | |
|  |  |  |  |  | |
| **N,% Inadequate Intake** |  |  |  |  | |
| **Vitamin A (equiv Retinol)** | 8 (2.5) | 1 (0.3)* | 6 (1.9) | 2 (0.6)* | |
| **Vitamin C** | 7 (2.2) | 0 (0.0)* | 6 (1.9) | 0 (0.0)* | |
| **Vitamin D** | 315 (100.0) | 315 (100.0) | 315 (100.0) | 315 (100.0) | |
| **Vitamin E** | 234 (74.3) | 132 (41.9)* | 227 (72.1) | 150 (47.6)* | |
| **Vitamin B1** | 0 (0.0) | 0 (0.0) | 0 (0.0) | 0 (0.0) | |
| **Vitamin B2** | 0 (0.0) | 0 (0.0) | 0 (0.0) | 0 (0.0) | |
| **Vitamin B3** | 0 (0.0) | 0 (0.0) | 0 (0.0) | 0 (0.0) | |
| **Vitamin B6** | 0 (0.0) | 0 (0.0) | 0 (0.0) | 0 (0.0) | |
| **Folate** | 8 (2.5) | 0 (0.0)* | 7 (2.2) | 0 (0.0)* | |
| **Vitamin B12** | 0 (0.0) | 1 (0.3) | 0 (0.0) | 0 (0.0) | |
| **Ca** | 57 (18.1) | 100 (31.7)* | 61 (19.4) | 81 (25.7)* | |
| **I** | 56 (17.8) | 99 (31.4)* | 64 (20.3) | 77 (24.4) | |
| **Fe** | 0 (0.0) | 0 (0.0) | 0 (0.0) | 0 (0.0) | |
| **P** | 0 (0.0) | 0 (0.0) | 0 (0.0) | 0 (0.0) | |
| **Mg** | 0 (0.0) | 0 (0.0) | 0 (0.0) | 0 (0.0) | |
| **Se** | 0 (0.0) | 0 (0.0) | 0 (0.0) | 0 (0.0) | |
| **Zn** | 3 (1.0) | 4 (1.3) | 5 (1.6) | 2 (0.6) | |
| **Cr** | 1 (0.3) | 0 (0.0) | 0 (0.0) | 1 (0.3) | |

*Prevalence of inadequacy has not been calculated for K and Na given that an EAR is lacking for both these micronutrients. Median intakes in all groups of both indices exceed the AI for K and Na, suggesting that risk of inadequate intake is low.*

*p for trend < 0.05

Estimates derived from generalized estimating equations with an identity link and exchangeable correlation structure (Gaussian family), using maternal ID as the clustering variable. The exposure was tertiles of adherence to the PHDI or PHDI-C, and the outcome was inadequate intake of each of the micronutrients listed above.
